# Exploring features and function of *Ss-riok-3*, an enigmatic kinase gene from *Strongyloides stercoralis*

**DOI:** 10.1186/s13071-014-0561-z

**Published:** 2014-12-05

**Authors:** Wang Yuan, Yingying Liu, James B Lok, Jonathan D Stoltzfus, Robin B Gasser, Weiqiang Lei, Rui Fang, Junlong Zhao, Min Hu

**Affiliations:** State Key Laboratory of Agricultural Microbiology, Key Laboratory of Development of Veterinary Diagnostic Products, Ministry of Agriculture, College of Veterinary Medicine, Huazhong Agricultural University, 1 Shizishan Street, Wuhan, 430070 China; Department of Pathobiology, School of Veterinary Medicine, University of Pennsylvania, 3800 Spruce Street, Philadelphia, PA 19104 USA; Department of Biology, Hollins University, Roanoke, VI 24020 USA; Faculty of Veterinary and Agricultural Sciences, The University of Melbourne, Corner of Flemington Road and Park Drive, Parkville, VI 3010 Australia

**Keywords:** Parasitic nematode, *Strongyloides stercoralis*, *riok-3*, Transgenesis

## Abstract

**Background:**

Right open reading frame protein kinase 3 (RIOK-3) belongs to the atypical kinase family. Unlike the other two members, RIOK-1 and RIOK-2, which are conserved from Archaea to humans, RIOK-3 occurs only in multicellular organisms. Studies on HeLa cells indicate that human RIOK-3 is a component of the 40S small ribosome subunit and supports cancer cell growth and survival. However, almost nothing is known about the function of RIOK-3. We explored the functional role of RIOK-3 encoding gene from *Strongyloides stercoralis*, a parasitic nematode of humans and dogs.

**Methods:**

To analyze the gene and promoter structure of *Ss-riok-3*, RACE-PCR and Genome-walker PCR were performed to isolate the full length cDNA, gDNA and promoter region of *Ss-riok-3*. RNA-seq was conducted to assess the transcript abundance of *Ss-riok-3* in different stages of *S. stercoralis*. Transgenesis was employed to determine the anatomic expression patterns of *Ss-riok-3*.

**Results:**

The RIOK-3 protein-encoding gene (designated *Ss-riok-3*) of *S. stercoralis* was characterized. The full-length complementary and genomic DNAs of the RIOK-3 encoding gene (*riok-3*) were isolated from this nematode. The cDNA of *Ss-riok-3* is 1,757 bp in length, including a 23 bp 5’-UTR, a 36 bp 3’-UTR and a 1,698 bp coding region encoding a protein of 565 amino acids (aa) containing a RIO kinase domain. RNA sequencing (RNA-seq) analysis revealed that *Ss-riok-3* is transcribed in all developmental stages of *S. stercoralis* assessed, with transcripts being particularly abundant in parasitic females. Gene structure analysis revealed that *Ss-riok-3* contains no intron. The putative promoter contains conserved promoter elements, including four TATA, two GATA, one inverse GATA and one inverse CAAT boxes. The promoter of *Ss-riok-3* drives GFP expression in the head neuron, intestine and body wall muscle of transgenic *S. stercoralis* larvae, and the TATA boxes present in the 3’-UTR of the gene immediately upstream of *Ss-riok-3* initiate transcription.

**Conclusions:**

The characterization of the RIOK-3 encoding gene from *S. stercoralis* provides a sound foundation for investigating in detail its function in the development and reproduction of this important pathogen.

**Electronic supplementary material:**

The online version of this article (doi:10.1186/s13071-014-0561-z) contains supplementary material, which is available to authorized users.

## Background

*Strongyloides stercoralis* is a parasitic nematode of humans and dogs, affecting estimated 60–100 million people worldwide and causing strongyloidiasis [1]. The life cycle of *S. stercoralis* is more complicated than those of most obligatory parasitic nematodes. Parasitic females (P Female) live in the intestine of the host and reproduce by mitotic parthenogenesis. They lay sexually differentiated eggs; these eggs hatch in the intestine, and post-parasitic first-stage larvae (PP L1) are either passed in the feces, or develop directly to auto-infective L3 (aiL3) within the host intestine. Then aiL3 invade somatic tissues and return to the intestine to produce a new generation of parasitic female adults (P Female). *S. stercoralis* undergoes low levels of autoinfection in immuno-competent hosts, however this process of autoinfection may proceed for sequential generations in an immuno-compromised host, with geometric expansion of parasite numbers and involvement of multiple body tissues, possibly leading to a fatal outcome [2]. In immune-competent hosts, PP L1 pass in feces from the host, and female PP L1 develop either homogonically to infective third-stage larvae (iL3) or heterogonically to free-living female adults (FL Female), whereas the male PP L1 develop to free-living male adults (FL Male) invariably. The FL Female and FL Male mate and produce embryos, which develop to iL3 through post free-living L1 (PFL L1); iL3 infect the host by skin penetration and develop to P Female [2].

The complex life cycle of *S. stercoralis* and lack of knowledge about its developmental biology has hampered the progress to control strongyloidiasis, requiring us to elucidate key biological processes in the development. Studies of the essential molecules that regulate the development of parasitic nematodes, including *S. stercoralis*, could facilitate the discovery of new interventions to control strongyloidiasis and other nematodiases.

One group of molecules essential in eukaryotic organisms is the protein kinases. Such kinases are key enzymes required for the regulation of a wide range of cellular processes, including cell-cycle progression, transcription, DNA replication and/or metabolic processes [3]. Based on their kinase domain sequences, protein kinases can be classified as eukaryotic protein kinases (ePKs) and atypical protein kinases (aPKs). In the human genome, 13 aPK families have been defined thus far, and one of them represents the family of the RIO protein kinases [4]. Four members of the RIO protein kinase family have been reported, i.e. RIOK-1, RIOK-2, RIOK-3 and RIOK-B. RIOK-1 and RIOK-2 are conserved from Archaea to humans, whereas RIOK-3 is only present in multicellular eukaryotes and, interestingly, RIOK-B is found in some bacteria [5]. RIOK-1, RIOK-2 and RIOK-3 contain a conserved RIO domain comprising multiple conserved functional motifs [5]. However, the sequences of these motifs differ among the three RIO kinases with the RIO domain of RIOK-1 and RIOK-3 being more similar in sequence. External to their RIO domain, RIOK-2 and RIOK-3 contain an N-terminal domain which is absent from RIOK-1 [6]. Consistent with these structural differences, the functions of the eukaryotic RIO kinases are also different. Although both RIOK-1 and RIOK-2 are involved in the maturation of the 40S ribosomal subunit including rRNA maturation and recycling other *trans-acting* factors involved in the maturation of 40S subunit, their functions therein are different, and they do not complement each other in yeast and human cells [7-9].

The function of RIOK-3 appears to diverge markedly from those of RIOK-1 and RIOK-2. In spite of the report that human RIOK-3 is also a component of 40S ribosomal subunit particles, its function in ribosomal synthesis is still unknown [10]. Human RIOK-3 is over-expressed in cancer cells [11-13] and interacts with caspase-10 to down-regulate NF-κB signaling by competing with receptor-interacting protein 1 (RIP1) and NF-κB-inducing kinase (NIK) [14]. These findings suggest that RIOK-3 is necessary for human cancer cell growth and survival [13-15]. In spite of the functional importance of RIOK-3 in human cells, nothing is known about the function of this molecule in nematodes, except for the early studies from RNAi screens in free-living nematode *Caenorhabditis elegans* [16-18], which have revealed no visible phenotypes including embryonic and larvae lethal, embryonic development variant, larvae developmental arrest, maternal sterile, organism morphology variant for the gene *Ce-riok-3* (data from WormBase, http://www.wormbase.org/#01-23-6). This information suggests that the function of *Ce-riok-3* in *C. elegans* is redundant. Whether *riok-3* function can be replaced and/or supported by other genes in parasites is still unknown.

Although RNAi is an efficient tool for the analysis of gene function in *C. elegans*, this approach does not work well in parasitic nematodes of animals studied to date [19]. Without effective RNAi-based methods, transgenesis constitutes an alternative approach for the functional genomic analysis of parasitic nematodes such as *S. stercoralis* [20-23]. In the present study, we explored RIOK-3 encoding gene *Ss-riok-3* of *S. stercoralis*, and investigated its temporal and spatial expression patterns towards the ultimate goals of uncovering its function and understanding its role in *S. stercoralis*.

## Methods

### Ethics statement

The *S. stercoralis* (UPD strain) was maintained in prednisolone-treated Beagles, in accordance with a protocol (permit no. SYXK-0029) approved by the Animal Ethics and Animal Experimentation Committee of Hubei Province. The care and maintenance of dogs were in strict accordance with the regulations for the Administration of Affairs Concerning Experimental Animals of P. R. China.

### Parasite maintenance and culture

The UPD strain of *S. stercoralis* was maintained in immune-suppressed dogs [24]; iL3 were collected from 7-10 day-old copro-cultures (22°C) *via* Baermann funnel and washed with sterile buffered saline (BU) [24-26]. Free-living adults of *S. stercoralis* were collected *via* Baermann funnel from copro-cultures after 2 days of incubation at 22°C and then were placed on nematode growth medium (NGM) plates and maintained on *Escherichia coli* OP50.

### Preparation of nucleic acids

Nucleic acids were isolated from 10,000–20,000 iL3s by a small-scale sodium proteinase K extraction [27] followed by purification using a mini-column (Wizard DNA Clean-Up System, Promega, USA). RNA was extracted from 20,000 ~ 30,000 iL3s using by TRizol reagent extraction (Life Technologies, USA). RNA yields were estimated spectrophotometrically (NanoDrop Technologies, Thermo Scientific, USA), and 1 μg of RNA was reverse transcribed to cDNA using a kit (Smart RACE Kit, Clontech, USA). Nucleic acids were stored at -80°C (RNA) or -20°C (DNA) until use.

### Isolating cDNA and promoter elements of *Ss-riok-3*

Two primers 1 F and 2R (Additional file 1) were designed to the sequences of three expressed sequence tags (ESTs) for *Ss-riok-3* (GenBank accession nos. BE224215, BE223756.1 and BG224329.1) from National Center for Biotechnology Information (NCBI, http://www.ncbi.nlm.nih.gov/). Using this primer pair, a region (255 bp) was PCR-amplified from cDNA transcribed from total RNA from iL3s of *S. stercoralis*, T/A-cloned into the vector pMD19T (Takara Biotechnology, Dalian, China) and directly sequenced. Based on the sequence obtained, gene-specific primers (3 F and 4R) were designed (Additional file 1). Using this pair of gene-specific primers and adaptor primers (from the RACE Amplification Kit, cat. no. 634923, Clontech), two partially overlapping cDNA fragments were produced from total RNA of *S. stercoralis* by 5’- and 3’-RACE, respectively. After sequencing the two partial cDNAs, three gene-specific primers (5 F, 6R and 8R) were designed to amplify the 5’- and 3’-termini of the *Ss-riok-3* cDNA (Additional file 1). The complete cDNA of *Ss-riok-3* was assembled using the sequences derived from the 5’-RACE and 3’-RACE amplicons and was searched and compared with the *S. stercoralis* genomic contigs (6 December 2011 draft; ftp://ftp.sanger.ac.uk/pub/pathogens/HGI/) by Geneious version 5.5.6 (http://www.geneious.com/). Subsequently, a pair of primers with restriction sites (Ss-riok3-*Eco*RI, Ss-riok3-*Hind*III) was designed to PCR-amplify the coding sequence (CDS) of *Ss-riok-3* using the Advantage 2 Polymerase Mix (Clontech), employing the following cycling conditions: initial 94°C, 5 min; then 94°C, 30 s, 55°C, 30 s; 72°C, 90 s for 30 cycles; final extension at 72°C, 10 min. The resultant amplicon was then (T/A) cloned into pMD19-T and sequenced.

To identify the *Ss-riok-3* promoter, four genomic DNA libraries were constructed using the Genome-Walker Kit (cat. no. 638904, BD Bioscience, Clontech) following the manufacturer’s instructions. Briefly, genomic DNA was digested with each of four restriction enzymes *Dra*I*, EcoR*V*, Pvu*II and *Stu*I (BD Biosciences, Clontech). Each of the four digested products was purified by phenol/chloroform extraction and then linked to an adapter with T4 DNA ligase. Then, PCR was performed using one adapter sense-primer and one gene-specific antisense-primer 10R (Additional file 1). All PCRs were conducted using the BD Advantage 2 Polymerase Mix (cat. no. 639201, Clontech) using the recommended touch-down PCR cycling protocol: 7 cycles at 94°C, 25 sec; 72°C, 3 min; 32 cycles at 94°C, 25 sec, 67°C, 3 min; final extension at 67°C for 7 min. The amplicons from all four genomic DNA libraries were examined separately on agarose gels; the largest products were purified employing TIANGEN Gel DNA purification kit (Tiangen Biotech, Beijing, China) and then (T/A) cloned into the vector pMD19-T (Takara Biotechnology) for subsequent sequencing.

To isolate the 3’ UTR of *Ss-riok-3* upstream gene (*Ss-rep-1*), the 3’-RACE was performed with one gene specific sense primer (Ss-rep1-3 F, Additional file 1) and the adapter primer (RACE Amplification Kit, Clontech). The sense primer Ss-rep1-3 F was designed based on the sequence obtained by Genome-Walker PCR. 3’-RACE PCR was performed using the Advantage 2 Polymerase Mix (Clontech) with following protocol: initial 94°C, 5 min; then 94°C, 30 s, 55°C, 30 s; 72°C, 90 s for 30 cycles; final extension at 72°C, 10 min. The PCR product was (T/A) cloned into pMD-19 T and sequenced. After confirming the end of the 3’ UTR of *Ss-rep-1* by aligning the cDNA with gDNA, two sense-primers, Ss-riok3pro1-*Hind*III and Ss-riok3pro2-*Hind*III and one anti-sense primer Ss-riok3pro-*Sma*I (Additional file 1) were designed to isolate the promoter region of *Ss-riok-3*, and the anti-sense primer Ss-riok3pro-*Sma*I was designed downstream (374 bp) of the start codon of *Ss-riok-3* and positioned according to the 91 nucleotides including a 42 bp artificial intron (see Additional file 2) between *Sma*I restrict site and the start codon of *gfp* of the promoter-less plasmid pAJ 02, in order to match the codon order. Each of the two sense-primers was used with their respective anti-sense primer to PCR-amplify 683 bp or 881 bp of genomic DNA upstream of *Ss-riok-3* (Additional file 2). The following cycling protocol was used: initial at 94°C for 3 min; then 94°C, 30 s; 72°C, 3 min for 5 cycles, followed by 94°C, 30 s; 67°C, 3 min for 25 cycles, and a final extension of 67°C, 7 min. Each amplicon was then (T/A) cloned into pMD19-T for sequencing.

### Bioinformatic analyses

The cDNA sequence of *Ss-riok-3* was compared with publicly available sequence data in non-redundant databases from NCBI (http://www.ncbi.nlm.nih.gov/) using BlastX [28]. The *Ss-riok-3* cDNA was translated into a predicted amino acid (aa) sequence (*Ss*-RIOK-3) using the program BioEdit (http://www.mbio.ncsu.edu/BioEdit/bioedit.html#downloads). Protein motifs of *Ss*-RIOK-3 were predicted by interrogating the databases PROSITE [29] (www.expasy.ch/tools/scnpsit1.html) and Pfam [30] (www.sanger.ac.uk/Software/Pfam/). The sequence of *Ss*-RIOK-3 and its homologs from selected species were aligned using the program MAFFT 7.0 [31] (http://mafft.cbrc.jp/alignment/software/). The functional domains and sub-domains were then identified in aligned RIOK3 sequences and then highlighted using the program Photoshop CS v.5.0.

The genomic sequence upstream of *Ce-riok-3* was retrieved from WormBase (WS243, http://www.wormbase.org/); promoter elements of *Ss-riok-3* and *Ce-riok-3*, and their respective upstream genes were predicted using the transcription element search system MatrixCatch (http://www.gene-regulation.com/cgi-bin/mcatch/MatrixCatch.pl) [32]. The two putative promoter sequences were aligned using MAFFT 7.0 [31].

For phylogenetic analysis, *Ss*-RIOK-3 was searched in the non-redundant database of NCBI (http://www.ncbi.nlm.nih.gov/) and 18 homologous sequences were retrieved from six nematodes [*Ascaris suum* (ERG84434.1), *Caenorhabditis brenneri* (EGT33672.1), *C. elegans* (NP_499173.1), *C. remanei* (XP_003113164.1), *Haemonchus contortus* (ADW27446.1), *Loa loa* (XP_003136543.1)]; two arthropods [*Aedes aegypti* (XP_001660046.1) and *Drosophila melanogaster* (AAF50965.1)]; nine vertebrates [*Canis lupus familiaris* (XP_005623064.1), *Danio rerio* (NP_001003614.1), *Homo sapiens* (NP_003822.2, EAX01152.1), *Mus musculus* (NP_077144.2), *Pan troglodytes* (XP_003953313.1), *Rattus norvegicus* (NP_001101893.1), *Salmo salar* (NP_001133982.1), *Xenopus laevis* (NP_001083392.1) and *X. tropicalis* (NP_001004996.1)]; and yeast [*Schizosaccharomyces pombe* (CAA15723.2)]. Sequences of these 18 RIOK-3 homologs were then aligned with that of *Ss-*RIOK-3 using Clustal X and manually adjusted. Phylogenetic analyses were conducted using the maximum likelihood (ML), maximum parsimony (MP) and neighbor-joining (NJ) methods based on the Jones-Taylor-Thornton (JTT) model in the software package MEGA v.5.0 [33]. Confidence limits were assessed by bootstrapping using 1,000 pseudo-replicates for ML, MP and NJ; other settings were obtained using the default values in MEGA v.5.0 [33]. A 50% cut-off value was implemented for the consensus tree.

### Transcriptional analysis

The *S. stercoralis* (PV001 line), derived from a single female worm of the UPD strain, was maintained as described previously [24,34-37]. Transcription profiles of *Ss-riok-3* were assessed in seven developmental stages of the PV001 line of *S. stercoralis*, including free-living females (FL Female), post-free-living first-stage larvae (PFL L1), infective third-stage larvae (iL3), *in vivo* activated third-stage larvae (L3+), parasitic females (P Female), post-parasitic first-stage larvae (PP L1) and post-parasitic third-stage larvae (PP L3). The parasites from different stages were isolated as described previously [34,35]. Transcription was quantified using RNA-seq [35]. Raw RNA-seq data for the seven developmental stages are available at http://www.ebi.ac.uk/arrayexpress/ under accession numbers E-MTAB-2192 (iL3) and E-MTAB-1164 (FL Female, PFL L1, L3+, P Female, PP L1, and PP L3). *Ss-riok-3*-specific transcript abundances were calculated as fragments per kilobase of coding exon per million fragments mapped (FPKM), and significant differences in FPKM values between developmental stages were determined using Cufflinks v.2.0.2 (http://cufflinks.cbcb.umd.edu/) [35,38-40]; *p*-values <0.05 were considered statistically significant, and *p*-values <0.001 were considered statistically highly significant. FPKM and 95% confidence intervals were plotted in Prism version 5.01 (GraphPad Software, Inc., http://www.graphpad.com/).

### Transformation constructs and transformation of *S. stercoralis*

To make reporter transgene constructs, alternative promoter regions of *Ss-riok-3* (Additional file 2), 683 bp and 881 bp, respectively, were digested with the endonucleases *Hind* III and *Sam* I (Thermo Fisher Scientific, USA) from the (T/A) cloned recombinant pMD19T plasmids and gel-purified using the Tiangen Gel Purification Kit (Tiangen Biotech). Each of the purified products was then sub-cloned into the promoter-less plasmid pAJ 02 [41], upstream of the *gfp* CDS, to create two plasmids: pRP2: *Ss-riok-3p* (683 bp)*::gfp::Ss-era-1* 3’-UTR and pRP4: *Ss-riok-3p* (881 bp)*::gfp::Ss-era-1* 3’-UTR (Additional file 2). The inserts of the plasmids were sequenced. After confirming the sequences, the two constructs were isolated using the TIANpure Midi Plasmid Kit (Tiangen Biotech) and each of them was diluted to 50 ng/μl and stored at -20°C. FL Females were transformed by gonadal micro-injection as previously described [23]. Briefly, solutions containing 50 ng/μL of plasmid pRP2 or pRP4 were injected into the gonads of FL Females. Transformed FL Females were then paired with one or two FL adult male(s) on an NGM + OP50 plates, and incubated at 22°C for subsequent egg laying. F1 post-free-living progeny were screened for fluorescence at 24, 48 and 72 h, respectively, after microinjection. *S. stercoralis* larvae were screened for expression of fluorescent reporter transgenes using a stereomicroscope (SZX12, Olympus) with epifluorescence. Then the *S. stercoralis* worms with GFP expression were placed on 2% agarose pad (Lonza, Basel, Switzerland), anesthetized using 50 mM levamisole (Sigma-Aldrich, USA) to examine in detail using a compound microscope (BX60, Olympus) with Nomarski Differential Interference Contrast (DIC) optics and epifluorescence. Larvae were imaged with a Spot RT Color digital camera and Spot Advanced image analysis software (Diagnostic Instruments Inc., USA). Captured images were processed using Photoshop CS v.5.0 as previously described [42]. Image-processing algorithms, limited to brightness and contrast adjustments, were all applied in linear fashion across the entire image.

## Results

### Characterization of *Ss-riok-3* cDNA

The full-length cDNA of *Ss-riok-3* is 1,757 bp in length comprising a 23 bp 5’-UTR, a 36 bp 3’-UTR and a 1698 bp coding sequence encoding 565 aa. A poly A tract, followed the 3’-UTR and the polyadenylation signal (AAUAAA) was found at the eighteenth nucleotide from the poly A tail. The predicted *Ss*-RIOK-3 protein exhibits low sequence identities (21.4–37.5%) to RIOK-3 s from various organisms, including other nematodes, fish, amphibians, vertebrates, with the highest identity (37.54%) being to *As*-RIOK-3 (ERG84434) from *A. suum*.

**Figure 1:**
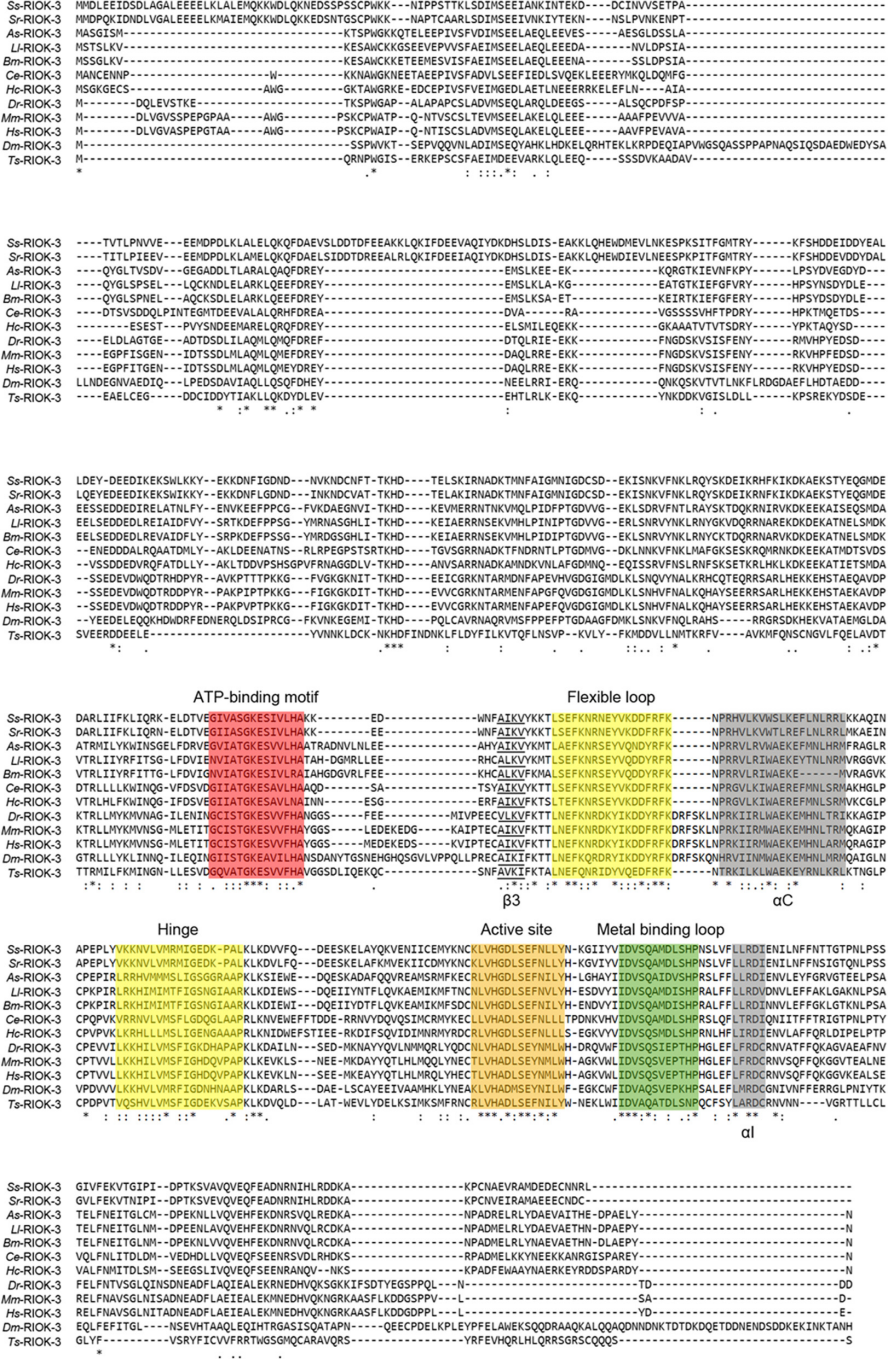
**Alignment of the inferred amino acid sequence of** ***Strongyloides stercoralis Ss*** **-RIOK-3 with those of RIOK-3 s from 12 other species of eukaryote.** The species selected were *Strongyloides ratti* (GCA_000208845.1, *Sr*-RIOK-3), *Ascaris suum* (ERG84434.1, *As*-RIOK-3), *Loa loa* (XP_003136543.1, *Ll*-RIOK-3), *Brugia malayi* (XP_001899758.1, *Bm*-RIOK-3), *Caenorhabditis elegans* (NP_499173.1, *Ce*-RIOK-3), *Haemonchus contortus* (ADW23594.1, *Hc*-RIOK-3), *Danio rerio* (NP_001003614.1, *Dr*-RIOK-3), *Mus musculus* (AAH33271.1, *Mm*-RIOK-3), *Homo sapiens* (NP_003822.2, *Hs*-RIOK-3), *Drosophila melanogaster* (AAF50965.1, *Dm*-RIOK-3), *Trichinella spiralis* (XP_003372228.1, *Ts*-RIOK-3). Alpha-helices (A-I) are highlighted in light grey; beta-sheet structures are marked under the alignment. Functional motifs, including the ATP-binding motif (red), the flexible loop (yellow), the hinge (yellow), the active site (orange) and the metal binding loop (green), are highlighted and labeled above the alignment. Identical residues are marked with asterisks.

The predicted aa sequence of *Ss*-RIOK-3 was aligned to homologs from 11 selected species, including seven nematode and four non-nematode species (Figure 1). The alignment showed that *Ss*-RIOK-3 contained a conserved RIO domain, including the ATP binding motif (P-loop), the flexible loop (which is unique in RIOK family), the hinge region, the active site and the metal binding motif (DFG-loop). The peptide sequence representing the ATP binding motif of RIOK-3 is STGKES. Interestingly, the ATP binding motif of RIOK-3 s from nematodes aligned here have Ala (A), instead of Ser (S), at the first position; in addition, Thr (T) was a Ser (S) at the second position in both *Ss*-RIOK-3 and RIOK-3 of *Strongyloides ratti* (*Sr*-RIOK-3). The predicted active site (catalytic loop) sequence of RIOK-3 s, LVH(G/A)DLSE(F/Y)N, also varies at the fourth aa and ninth aa among distinct species. With the exception of *Trichinella spiralis* (*Ts*-RIOK-3), the parasitic nematodes studied herein have a Gly (G) at the fourth position instead of Ala (A), as seen in fish, mammals and the free-living nematode *C. elegans*. Another aa alteration occurred at the ninth aa in the predicted active site; all nematodes selected here have a Phe (F), whereas the RIOK-3 s from fish, insects and mammals have a Tyr (Y) at this position. The Asp (D) at the fifth position in the active site, which is associated with kinase activity, is identical among the species studied and is conserved across known ePKs [3,43]. The sequence alignment (Figure 1) also highlights that *Ss*-RIOK-3 and *Sr*-RIOK-3 contain a larger N-terminal region compared with RIOK-3 s from other species studied here. This divergence in the N-terminus reflects the low identity between *Strongyloides* RIOK-3 s and their homologs in other taxa.

### Evolutionary relationship of the predicted protein *Ss*-RIOK-3 to homologs from other species

**Figure 2:**
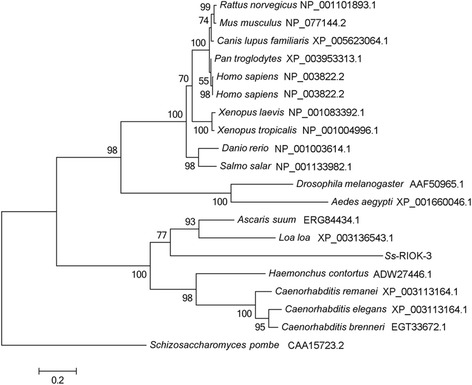
**Phylogenetic relationship of** ***Strongyloides stercoralis Ss*** **-RIOK-3 with homologs from 18 selected species of eukaryote.** These species include six nematode species, two insects, two fish, two amphibians and five mammals. The RIOK-3 from *Schizosaccharomyces pombe* (CAA15723.2) is used as an outgroup. GenBank accession numbers of sequences are listed beside the species name. Bootstrap values are displayed above or below the branches.

Results of phylogenetic analyses revealed that there is concordance in topology among ML, MP and NJ trees (Figure 2). RIOK-3 s from seven nematodes, including *Ss*-RIOK-3, grouped together with high nodal support (100%). *Ss*-RIOK-3 had the closest relationship to the RIOK-3 s from parasitic nematodes of clade III [44] with moderate support (77%). Another large cluster containing RIOK-3 s from six mammals, two amphibians, two fish and two insects grouped together with high bootstrap support (98%). RIOK-3 s from two species of amphibian and two species of fish grouped with the RIOK-3 s from six mammalian species with strong support (100%). RIOK-3 s of two insect species grouped together with strong nodal support (100%).

### Characterisation of the organization of the gene *Ss-riok-3*

**Figure 3:**
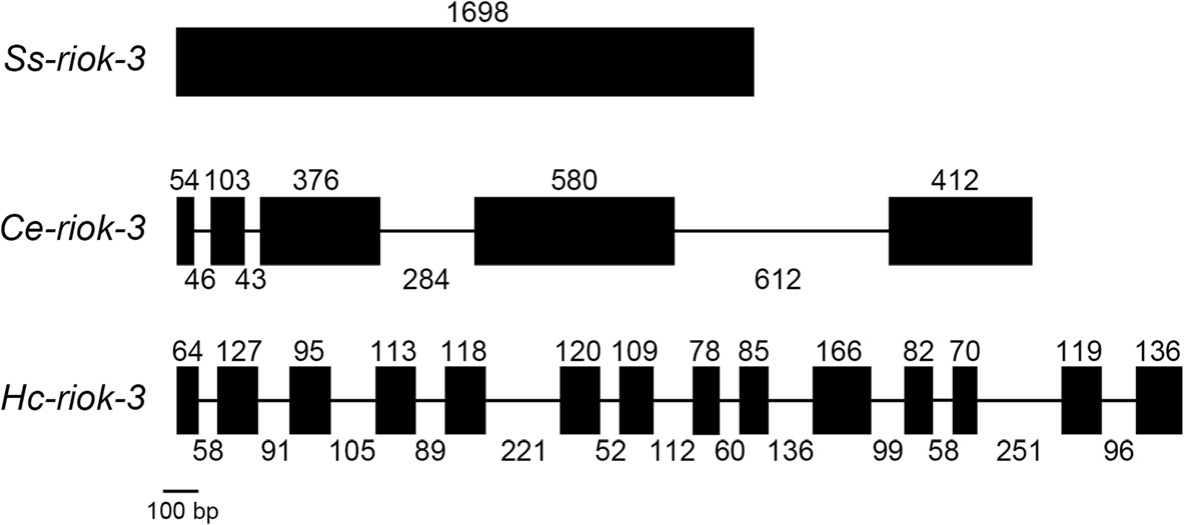
**The gene structure of** ***Strongyloides stercoralis Ss*** **-** ***riok*** **-** ***3*** **compared with its homologs from** ***Caenorhabditis elegans*** **and** ***Haemonchus contortus*** **.** Black boxes indicate exons, with the numbers above indicating exon lengths. Introns are indicated by lines between the exons, with numbers indicating intron lengths.

Comparison of cDNA and genomic DNA sequences of *Ss-riok-3* revealed that there are no introns in the coding sequence of this gene or in its untranslated regions (Figure 3). Comparison of gene structures between *Ss-riok-3* and two homologs, *Ce-riok-3* from *C. elegans* (ZK632.3 from WormBase) and *Hc-riok-3* from *H. contortus* [45] showed that *Ce-riok-3* contains five exons of 54–580 bp in length and four introns of 43–612 bp, and that *Hc-riok-3* contains 14 exons of 64–166 bp in length and 13 introns of 52–251 bp.

### Analysis of the predicted promoter region of *Ss-riok-3*

**Figure 4:**
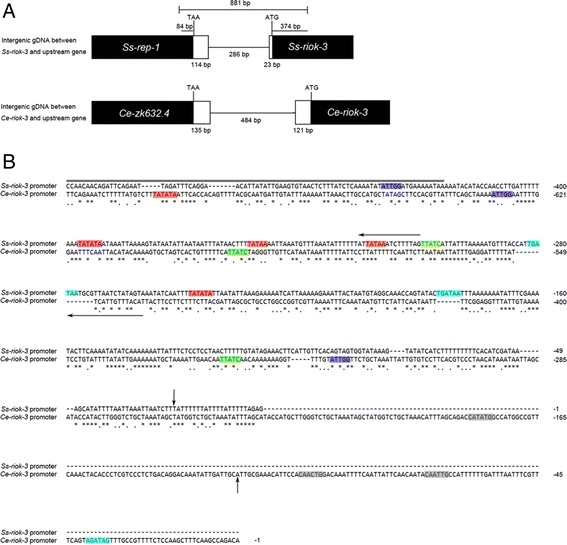
**Diagram of upstream gene and intergenic regions of the genes** ***Ss-riok-3*** **(** ***Strongyloides stercoralis*** **) and** ***Ce-riok-3*** **(** ***Caenorhabditis elegans*** **), and alignment of promoter elements predicted from the upstream genomic DNA sequences of these two genes. (A)** The upstream gene and intergenic regions. Black boxes indicate partial coding sequence, with gene name indicated; a white box indicates an untranslated region (UTR); a line indicates an intergenic region. Termination and initiation codons are marked on the coding strand, and UTRs and intergenic regions marked below white boxes and the lines, respectively. Brackets indicate genomic DNA inserted into the transformation constructs. **(B)** Alignment of the predicted promoters of *Ss-riok-3* and *Ce-riok-3*. Genomic DNA sequences upstream of the initiation codon of *Ss-riok-3* and *Ce-riok-3* were aligned. Colored boxes represent the promoter elements: CAAT (CCAAT) or inverse CAAT (ATTGG) motif (blue), inverse GATA (TTATC) (green), GATA (WGATAR) (turquoise), E- (CANNTG) (grey) and TATA (red) boxes. The numbers represent the positions of the nucleotides upstream of the start codon. The thick black line above the *Ss-riok-3* promoter indicates the 84 bp 3’-end of the coding sequence of *Ss-rep-1*. Left arrows above *Ss-riok-3* and below *Ce-riok-3* indicate the 3’-UTRs of the *Ss-rep-1* and *Ce-zk632.4*. Vertical arrows indicate the transcription start site.

Analysis of the 5’-flanking genomic region (1,600 bp in length) revealed that the gene immediately upstream of *Ss-riok-3* is *Ss-rep-1,* the homolog *of Ce-rep-1* (NM_001027757.3) encoding isoform a of *C. elegans* Rab escort protein (REP-1), which mediates Rab prenylation by binding with Rab proteins to regulate vesicular trafficking in cells [46-49]. The intergenic region between the termination codon of *Ss-rep-1* and the start codon of *Ss-riok-3* is 423 bp in length, and contains the 114 bp 3’-UTR of *Ss-rep-1* and the 23 bp 5’-UTR of *Ss-riok-3* separated by 286 bp of genomic DNA (Figure 4A). This region is A + T rich with a content of 79%. *Ce-riok-3* is the downstream gene of operon CEOP3628 in *C. elegans*. Its upstream gene is *Ce-ZK632.4*, which encodes the homolog of human phosphomannose isomerase. The genomic region between *Ce-riok-3* and *Ce-ZK632.4* is 740 bp long, with an A + T content of 68.4%. The putative promoter region of *Ss-riok-3*, showed limited similarity to the 5’-flanking region of *Ce-riok-3* (Figure 4B). The search for promoter elements identified four TATA, two GATA, one inverse GATA and one inverse CAAT boxes for *Ss-riok-3.* By contrast, the predicted promoter region of *Ce-riok-*3 contained one TATA, one GATA, two inverse GATA, three E-boxes and two inverse CAAT boxes. Three of the four TATA boxes in the promoter region of *Ss-riok-3* are positioned in the 3’-UTR region of *Ss-rep-1*. The single TATA box in the 5’-flanking region of *Ce-riok-3* is similarly situated in the 3’-UTR region of *Ce*-ZK632.4. The four nucleotides preceding the start codon are AGAG in *Ss-riok-3* and GACA in *Ce-riok-3*. Both of these sequences differ from the four nucleotides, AAAA, that commonly precede the start codons of protein-encoding genes in *C. elegans* [50].

### Transcriptional analysis of *Ss-riok-3* in different developmental stages

**Figure 5:**
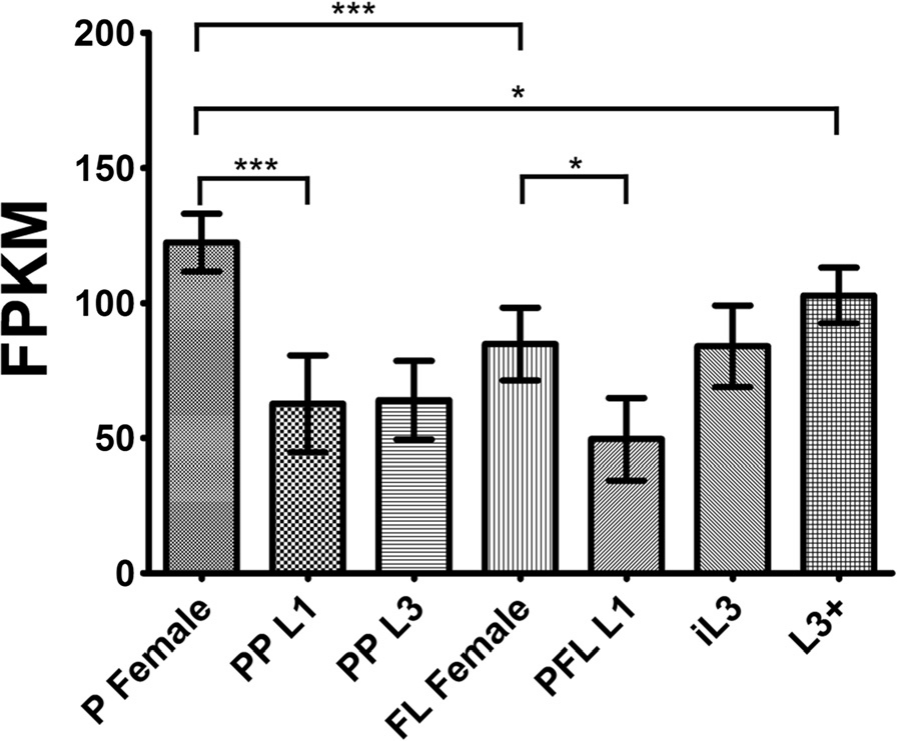
**Transcriptional profiles for** ***Strongyloides stercoralis Ss-riok-3*** **.** Transcript abundances were determined for the complete coding region of *Ss-riok-3* for seven distinct developmental stages: parasitic females (P Female), post-parasitic first-stage larvae (PP L1), post-parasitic third-stage larvae (PP L3), free-living females (FL Female), post free-living first-stage larvae (PFL L1), infective third-stage larvae (iL3), and in vivo activated third-stage larvae (L3+). Transcript abundance was calculated as fragments per kilobase of coding exon per million mapped reads (FPKM). Brackets with one star represent significant differences with a statistical probability of *p* < 0.05. Brackets with three stars represent the significant differences with statistical probability of *p* < 0.001. Error bars represent 95% confidential intervals.

*Ss-riok-3*-specific transcripts were detected in all developmental stages of *S. stercoralis* assayed, with the highest abundance in P Females [please check whether P Female is used as a singular or plural noun throughout MS] and the lowest abundance in the PFL L1 (Figure 5). Notably, the abundances of *Ss-riok-3* transcripts were higher in the parasitic stages of *S. stercoralis*, including P Female and L3+, than in the free-living stages, including PP L1, PP L3, FL Female, PFL L1 and iL3. *Ss-riok-3* transcript abundance was significantly (*p* < 0.001) higher in P Females than in PP L1. A less marked, but significant (*p* < 0.05) increase in *Ss-riok-3* transcript abundance was observed between L3+ and P Females. The abundance of *Ss-riok-3* transcripts was minimal in PFL L1 and significantly lower than in parental FL Females (*p* < 0.05). There was a generally an increasing trend in *Ss-riok-3* transcript abundance during development from PFL L1 to iL3 in the external environment, and in the further development within the host to the L3+ and then to the P Female. Although both parasitic and free-living females contained high levels of *Ss-riok-3* mRNA, the abundance of these transcripts was significantly (*p* < 0.001) higher in P Females than in FL Females.

### Anatomic expression pattern

**Figure 6:**
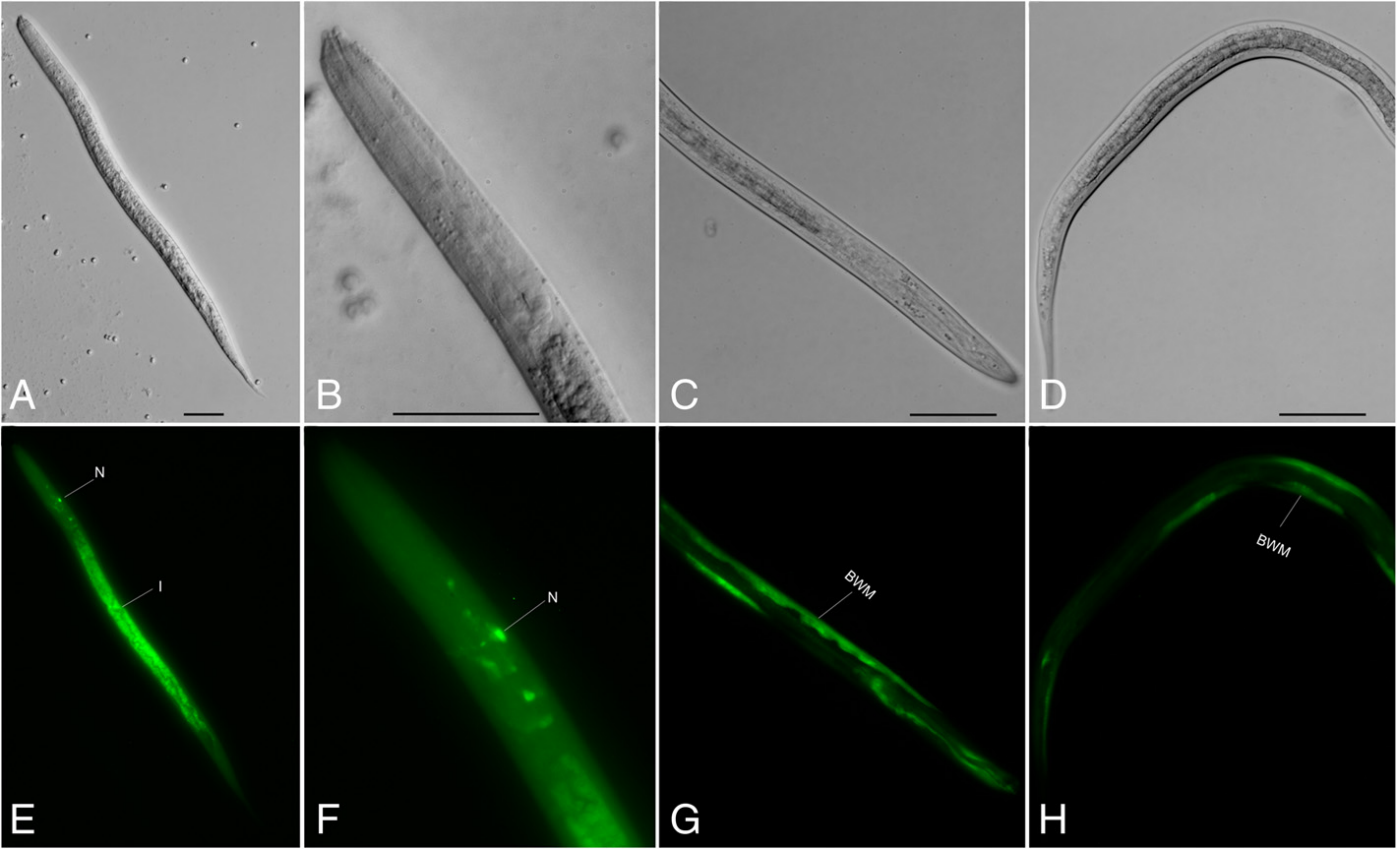
**The anatomical expression pattern of** ***Ss-riok-3*** **in the post-free-living first and second stage larvae and infective third stage larvae of** ***Strongyloides stercoralis*** **.** DIC **(A,B)** and fluorescence **(E,F)** images of transgenic *S. stercoralis* post-free-living L1-L2 stage larvae expressing *Ss-riok-3p::gfp::Ss-era-1 t*. **(A,E,)**; GFP expression in the intestine (i) and head neuron (N). DIC **(C,D)** and fluorescence (G, H) images of transgenic *S. stercoralis* infective third stage larvae expressing *Ss-riok-3p::gfp::Ss-era-1 t*. **(G,H)** GFP expression in the body wall muscle (BWM; white arrows). Scale bars = 50 μm.

The anatomic expression pattern of *Ss-riok-3 in vivo* was inferred from those of *gfp* expression under control of the 881 bp putative promoter in larval *S. stercoralis* transformed with pRP4 (Additional file 2); 48 h after transformation, the PFL L1 - L2s expressed GFP in the head neuron and intestine (Figure 6E,F). By contrast, GFP expression under the *Ss-riok-3* promoter predominated in body muscle of iL3s transformed with pRP4 (Figure 6G,H). Worms transformed with pRP2, which includes a 683 bp putative *Ss-riok-3* promoter, did not express GFP (data not shown). No fluorescence was observed in non-transgenic worms.

## Discussion

RIOKs are atypical protein kinases, with the first representative (RIOK-1) discovered in yeast in 1997 [51]. All species contain at least two members of RIOK (i.e. RIOK-1 and RIOK-2), but multicellular, eukaryotic organisms studied to date also possess RIOK-3 [5]. In human cells and yeast, RIOK-1 and RIOK-2 play important roles in the process of ribosomal biology, including recycling of the trans-acting factors and/or rRNA cleavage [7-9,52]. Compared with RIOK-1 and RIOK-2, much less is known about RIOK-3. The function of RIOK-3 is different from those of RIOK-1 and RIOK-2 in human cells, with RIOK-3 controlling tumor cell invasiveness, suppressing the activity of NF-κB and supporting type I-interferon production [13,14,53,54]. Recently, human RIOK-3 was discovered in ribosomal 40S subunit particles [10].

Currently, nothing is known about the function of RIOK-3 in nematodes. We have begun to rectify this by investigating the structure, transcription patterns and anatomic expression of *Ss-riok-3*, the RIOK-3-encoding gene from *S. stercoralis*. We identified only one transcript of *Ss-riok-3*; however, in *C. elegans*, there are two transcript variants (isoforms) of *Ce-riok-3*, with the same coding sequence but with 5’-UTRs of differing lengths (http://www.wormbase.org/). The human genome also encodes an additional *riok-3* transcript variant, but the relevant locus is predicted to be a pseudo-gene [4]. Consistent with our findings for *S. stercoralis*, *riok-3* genes of other parasitic nematodes, including *A. suum*, *Dirofilaria immitis* and *H. contortus*, appear to produce only one transcript.

All four reported members from the RIO protein kinase family (i.e. RIOK-1, RIOK-2, RIOK-3 and RIOK-B) contain the conserved RIO domain, including an ATP binding motif, a flexible loop, a hinge, an active site and a metal-binding motif [5]. The RIO domain is similarly conserved in *Ss*-RIOK-3. Like the RIOK-1 s and RIOK-2 s, RIOK-3 s also lacks the substrate-binding motif present in ePKs, but has an inserted flexible loop [3,5]. It is interesting that the predicted peptide sequence of the ATP-binding motif of *Ss-*RIOK-3 has two aa substitutions compared with those of the other species studied herein. For example, the sequence is ASGKES in *S. stercoralis* and *S. ratti*, ATGKES in the other nematodes and STGKES in the non-nematode species selected. Analyses of RIOK-1 and RIOK-2 in a complex with ATP [6,55] reveal that the putative ATP binding motif, along with the beta strand-three, actually participate in binding ATP and that the Lys (K) located in the beta strand-three is required for this interaction. Site mutation changing the Lys (K) in the beta strand-three to Ala (A) in the ATP binding motifs of RIOK-2 and RIOK-3 in yeast and human cells ablate the ATP binding activity and affect the kinase functions of these enzymes [54,56]. Although the Lys (K) from the ATP binding loop does not directly interact with ATP, it is essential for RIOK-2 to bind with pre-40S subunit to participate in ribosomal biogenesis [56]. Whether the aa substitutions in the ATP-binding motif of *Ss*-RIOK-3 influence its affinity for ATP or AMP is unknown and warrants future investigation.

An alignment of predicted aa sequences (Figure 1) also revealed that RIOK-3 s from selected species, including *Ss*-RIOK-3, contain a N-terminal region that is much larger than the N-terminal regions of RIOK-1 s and RIOK-2 s. This finding is consistent with a comparative analysis of the predicted structures for RIOKs from *C. elegans* and parasitic nematodes for which extensive genomic and transcriptomic data sets are available [57]. The precise function of this N-terminal region of RIOK-3 s is unknown; however, findings for RIOK-3 in human cells support that the N-terminal region of this RIOK-3 is involved in the binding of polyubiquitin and the suppression of TNFα-mediated NF-κB activity [14,53]. In addition, bioinformatic analysis predicts that the N-terminal region of *Ce*-RIOK-3 is involved in transmembrane transport [57]. In the present study, the *Strongyloides* RIOK-3 s (*Ss*-RIOK-3 and *Sr*-RIOK-3) contained a larger N-terminus than RIOK-3 s from other selected species; this largely reflects the low sequence identity between *Strongyloides* RIOK-3 and its homologs. This difference suggests that the N-terminus of *Ss*-RIOK-3 might have a unique function.

The analysis of the *S. stercoralis* transcriptome reveals that the transcription of *Ss-riok-3* is high in the parasitic life stages of this worm, with the highest level in P Females. It also shows that, with the exception of iL3, transcripts of *Ss-riok-3* are more abundant in FL Females than in post parasitic and post free-living larvae. The abundance of *Ss-riok-3* transcripts in female worms suggests that *Ss-riok-3* plays a functional role in reproduction. Due to the absence of transcripts from FL Males, we could not compare transcript abundance between FL Females and FL Males. However, as *S. stercoralis* is parthenogenetic, there is no male adult within the host. Nonetheless, the peak transcript abundance in P Females still suggests a key function for *Ss*-RIOK-3 in this developmental stage. In other parasitic nematodes (*A. suum*, *B. malayi* and *H. contortus*) *riok-3* is transcribed at high levels in eggs but does not show significant differences between female and male adults in these three parasites [57]. This information is consistent with trends of *riok-3* transcription in embryos of *C. elegans* and parasitic nematodes, supporting the proposal of constitutive expression of this gene during embryogenesis [57,58].

Notably, *Ss-riok-3* transcription is also enriched in L3+, which have invaded the host and resumed development to the P Female stage. The abundance of *Ss-riok-3* transcript in the parasitic stages of *S. stercoralis* might indicate that *Ss*-RIOK-3 participates in sexual maturation of L3s developing to P Females. In *C. elegans*, analysis of gene expression cluster during sexual differentiation revealed that *Ce-riok-3* is enriched in mature hermaphrodites [59]. This trend is echoed by the enrichment of *Ss-riok-3* transcripts in the P Females and FL Females of *S. stercoralis*, suggesting its possible roles in female reproductive biology of this nematode. The analyses of transcript abundance did not show transcriptional difference between FL Females and iL3, however, the promoter activity also shifts from intestine in PFL L1 to body wall muscle in iL3, which suggest that *Ss*-RIOK-3 could be involved in maintaining the activity of body wall muscle of iL3 to support host-finding and infection.

*Ce-riok-3* is present in an operon (CEOP3628) [60]. A similar genomic context is not supported for *Ss-riok-3*, as the donor SL1 and SL2 snoRNA is absent from the 5’ end of *Ss-riok-3* cDNA, indicating no association with an operon; the gene immediately upstream of *Ss-riok-3* (*Ss-rep-1*) is not found in the 5’-flanking region of *Ce-riok-3*. The intergenic region between the stop codon of *Ss-rep-1* and the start codon of *Ss-riok-3* is 423 bp. In the initial gene localization analysis, the 309 bp genomic DNA, comprising 23 bp of 5’-UTR of *Ss-riok-3* and the 286 bp of intergenic region between the start codon of *Ss-riok-3* and the end of 3’-UTR of *Ss-rep-1* (Additional file 2), was predicted to be the promoter region of *Ss-riok-3* and was thus cloned into the reporter gene plasmid to make a transformation construct pRP2 (Additional file 2). However, this region did not drive GFP expression in PFL L1-L2s (data not shown). Only the transformation construct (pRP4) comprising 423 bp genomic DNA region, including the 23 bp of 5’-UTR of *Ss-riok-3*, the 286 bp of intergenic genomic DNA and the 114 bp of 3’-UTR of *Ss-rep-1*, drove strong GFP expression in the intestine and head neuron of PFL L1s and L2s of *S. stercoralis*. The analysis of the potential promoter regions of *Ss-riok-3* revealed that three of the four TATA boxes in the 5’-flanking region of *Ss-riok-3* are actually located in the 3’-UTR of *Ss-rep-1* which is immediately upstream of *Ss-riok-3*. Failure of the 683 bp promoter element in pRP2, which lacks these three TATA boxes, to drive *gfp* expression and the contrasting, robust reporter expression under control of the 881 bp promoter element in pRP4, which includes them, demonstrates that, despite their location within the regulatory sequences of another gene, the three TATA boxes in the 3’-UTR of *Ss-rep-1* are essential for initiating transcription of *Ss*-*riok-3 in vivo*. The TATA box is a common promoter element, which is recognized by RNA polymerase II during transcription initiation in most protein-coding genes [61,62]. Also present in the putative *Ss-riok-3* promoter are four GATA and two inverse GATA boxes. The GATA transcription factor (TF) family consists of GATA 1 to 6 that contain zinc finger motif capable of binding to target DNA [63]. GATA transcription factors regulate gene expression and play important roles in eukaryotic development [64-68]. In *C. elegans*, GATA TFs regulate gut-specific gene transcription, and ELT-2 TF predominantly controls the development of the intestine [69,70]. The present results showed that the sequences of GATA boxes (TGATAA) in the predicted promoter region of *Ss-riok-3* are consistent with ELT-2 binding sequence in *C. elegans*, which indicate GATA TFs regulate *Ss-riok-3* transcription and expression in the intestine of PFL L1s.

The activity of the *Ss-riok-3* promoter in the intestine of PFL L1-L2s is consistent with results of a high-throughput *in vivo*-analysis of gene expression in *C. elegans*, revealing that *Ce-riok-3* is expressed in the intestine of larvae and adults [71,72]. In *C. elegans*, the intestine is a relatively large and important organ whose functions include not only digestion of food and absorption of nutrients, but also synthesis and storage of macromolecules, initiation of innate immune response to pathogens as well as the production of egg yolk protein [73-76]. In *C. elegans*, the intestine develops from a single cell lineage, beginning with progenitor cell E. During this development, GATA TFs regulate and activate intestine-specific gene expression which supports intestinal differentiation and maintenance [75,77]. Distinctive localization of *Ce-riok-3* to larval and adult intestine and its enrichment in hermaphrodites [59] could indicate that *Ce*-RIOK-3 supports intestinal development and function, and suggests that RIOK-3 plays a role in the reproductive biology of *C. elegans*. Similarly, the abundance of *Ss-riok-3* transcripts in P Females and FL Females of *S. stercoralis* as well as the intestinal localization of *Ss-riok-3* promoter activity suggest that *Ss*-RIOK-3 supports intestinal function during development from larvae to adults, and possibly reproductive functions in adult females of this parasitic nematode.

In addition to the intestine of *S. stercoralis*, the head neuron of PFL L1 - L2s also had GFP expression that is unique to *S. stercoralis* compared with expression pattern for *Ce-riok-3* in *C. elegans*, indicating a functional difference between *Ss*-RIOK-3 and *Ce*-RIOK-3, and could suggest central and distinctive roles for RIOK-3 in the *S. stercoralis*, which warrant further investigation. Also, the shift of *Ss-riok-3* promoter activity from intestine in PFL L1-L2s to body wall muscle in iL3s, which is also found in the promoter activity of *Ss-riok-1* from neurons in PFL L1 to body wall muscle in iL3 [42], suggests a remolding of tissues in iL3 to prepare the parasite for infecting the host animal [2].

## Conclusions

In conclusion, we have identified the RIOK-3 encoding gene *Ss-riok-3* in *S. stercoralis. Ss*-RIOK-3 is predicted to contain conserved RIO domain with two aa substitutions in the ATP-binding motif compared with RIOK-3 s from other nematodes, and from other selected species of invertebrates and vertebrates. *Ss-riok-3* transcripts are most abundant in the parasitic stages of *S. stercoralis*, and the *Ss-riok-3* promoter is active in the intestine and some cephalic neurons of PF L1-L2s, suggesting that *Ss-riok-3* supports the development of the intestine of larvae and could be involved in the reproductive biology of female adults. The shift of *Ss-riok-3* promoter activity during the development of *S. stercoralis* from PFL L1 to iL3 suggests a potential role of *Ss-riok-3* in morphogenesis of iL3. The finding that the TATA boxes located in the 3’-UTR of *Ss-rep-1*, the gene immediately upstream of *Ss-riok-3*, are essential regulatory elements to initiate the transcription of *Ss-riok-3* also underscores the functional overlap of regulatory sequences in genes that are closely linked in the *S. stercoralis* genome. Whether *Ss*-RIOK-3 participates in the reproductive biology in female adults still needs further investigation.

## Additional files

Additional file 1:
**Codes and DNA sequences of oligonucleotide primers used in the present study.** These primers were employed for the isolation of 3’ cDNA of *Ss-rep-1*, cDNAs and predicted promoter regions of *Ss-riok-3* using PCR-based approaches and for the construction of plasmids for transgenesis. Additional file 2:
**Diagram of** ***Ss-riok-3*** **transcriptional reporter construct pRP2 and pRP4 used to transform.** *Strongyloides stercoralis*. The promoter structure of *Ss-riok-3* is shown. (A) Black box represents a coding region of the gene. White box represents the un-translated region. Line represents intergenic region. The lengths of intergenic region, untranslated region, regions that were cloned into the pAJ 02 vector are marked above or below each corresponding region indicated with brackets. (B) The *Hind*III and *Sma*I restrict sites along with the start codon of *gfp* were marked. The length of sequence with an artificial intron between the *Sma*I restrict site and *gfp* start codon is indicated. The 683 bp and 881 bp regions (marked below and above the promoter structure, respectively) between *Ss-rep-1* and *Ss-riok-3* were separately inserted into the vector pAJ02 [41], creating plasmids pRP2 and pRP4, respectively. Length of *gfp* with artificial introns and *Ss-era-1* 3’-untranslated region (UTR) in the constructs are indicated.
