# Supplementary material for: Exploring features and function of Ss-riok-3, an enigmatic kinase gene from Strongyloides stercoralis
Source: Parasit Vectors. 2014 Dec 5;7:561. doi: 10.1186/s13071-014-0561-z (PMC4265397; doi:10.1186/s13071-014-0561-z)
Supplement: Additional file 1: — Codes and DNA sequences of oligonucleotide primers used in the present study. These primers were employed for the isolation of 3’ cDNA of Ss-rep-1, cDNAs and predicted promoter regions of Ss-riok-3 using PCR-based approaches and for the construction of plasmids for transgenesis. [file 13071_2014_561_MOESM1_ESM.doc]

Additional file 1

| Primer names | Primer sequence |
| --- | --- |
| 1F | CAACTTATGAACAGGGTATGGATGAAG |
| 2R | GACTCCATACTTTTAATACATGACGTGG |
| 3F | CCGTTGAAGGTATAGTAGCTAGTGG |
| 4R | GCATTAATTTGAGCTTTCTTTAAACGGCG |
| 5F | CCTGATCTAAAACTAGCATTAGAAC |
| 6R | CTTCATCCATACCCTGTTCATAAGTTG |
| 8R | CATCAGAACAATCTGGAATATTCATACC |
| 10R  20R  Ss-riok3-*EcoR*I  Ss-riok3-*Hind*III  Ss-rep1-3F  Ss-riok3pro1-*Hind*III | CATCATACTCATCTAACAACGCTTC  GCAATTTCCTCACTCATAATATCTG  CGGAATTCATGATGGATCTAGAGGAAATAG  CCCAAGCTTTTACAATCTATTATTACATTC  ATGGGCAAACTGCACCTAAAGGT  AAGCTTTTATCATTATTTAAAAATGTTTACC |
| Ss-riok3pro2-*Hind*III | AAGCTTCCAACAACAGATTCAGAATTAGATTTC |
| Ss-riok3pro-*Sma*I | CCCGGGTCGGCTTCTTCAAAATCAG |
