# Supplementary figures and images for: Exploring features and function of Ss-riok-3, an enigmatic kinase gene from Strongyloides stercoralis
Source: Parasit Vectors. 2014 Dec 5;7:561. doi: 10.1186/s13071-014-0561-z (PMC4265397; doi:10.1186/s13071-014-0561-z)

Additional file 2


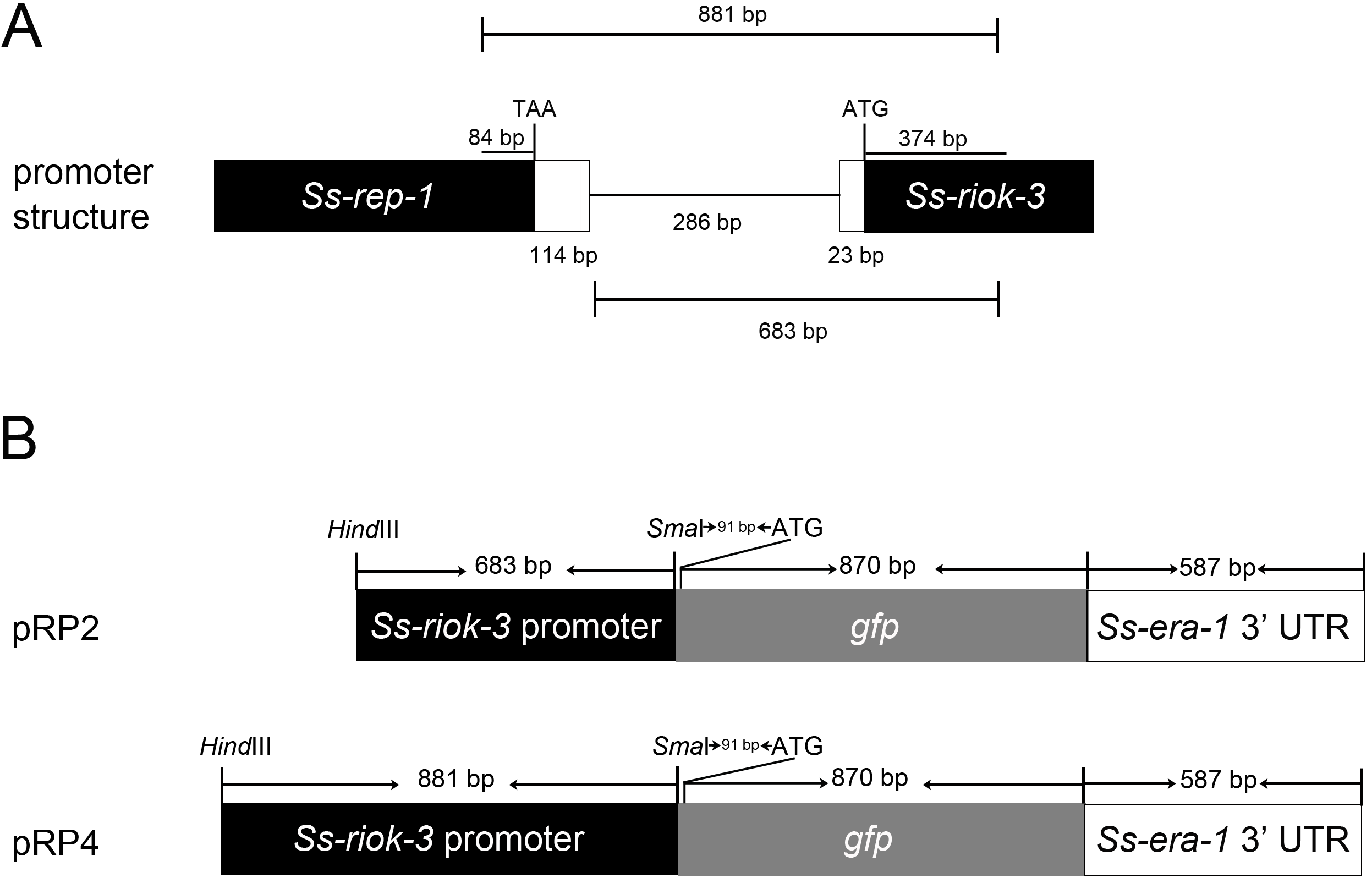

Supplement: Additional file 2: — Diagram of Ss-riok-3 transcriptional reporter construct pRP2 and pRP4 used to transform. Strongyloides stercoralis. The promoter structure of Ss-riok-3 is shown. (A) Black box represents a coding region of the gene. White box represents the un-translated region. Line represents intergenic region. The lengths of intergenic region, untranslated region, regions that were cloned into the pAJ 02 vector are marked above or below each corresponding region indicated with brackets. (B) The HindIII and SmaI restrict sites along with the start codon of gfp were marked. The length of sequence with an artificial intron between the SmaI restrict site and gfp start codon is indicated. The 683 bp and 881 bp regions (marked below and above the promoter structure, respectively) between Ss-rep-1 and Ss-riok-3 were separately inserted into the vector pAJ02 [41], creating plasmids pRP2 and pRP4, respectively. Length of gfp with artificial introns and Ss-era-1 3’-untranslated region (UTR) in the constructs are indicated. [file 13071_2014_561_MOESM2_ESM.doc]
